# The need for ecological momentary assessment in researching emotional factors in language education

**DOI:** 10.3389/fpsyg.2023.1115871

**Published:** 2023-04-18

**Authors:** Xiaodong Li

**Affiliations:** School of Foreign Studies, University of Science and Technology Beijing, Beijing, China

**Keywords:** emotional variables, ecological momentary assessment, emotions, second language acquisition, classroom learning, dynamic process

## Abstract

Language learning is an emotional and dynamic process, which is marked by fluctuations in language learners’ positive and negative emotional variables (e.g., boredom, enjoyment, anxiety). Presumably, evidence can be found for an ecological view of the patterns and variations involved in language learners’ emotions under the influence of the interactive individual and contextual elements of classroom learning. The present study contends that an ecological momentary assessment (EMA), which is compatible with the complex dynamic system theory (CDST) can help to explore the dynamics of language learners’ emotional variables as they develop out of the process of classroom language learning. EMA is capable of tracing the moment-by-moment changes in a certain emotional trait in language learners as they are learning a foreign or second language. This innovative approach to research compensates for the shortcomings of retrospective studies (the delay of recalls) and also single-shot research designs (for data collection). It is fit for the assessment of the emergent patterns of L2 emotional variables. The distinctive features and pedagogical implications will be further discussed here.

## Introduction

Ecological momentary assessment (EMA) was an attempt originating from clinical psychology to substitute or complement static retrospective works of research, which failed to capture how behavior could change through the passage of time and from one context to another (Hektner et al., 2007). EMA is not considered an individual method of research; rather, it includes an array of methods and methodological approaches. It encompasses a repeated sampling of the research participants’ behaviors and experiences at the current time in real life or education and in the participants’ natural contexts. EMA seeks to increase ecological validity, lower recall bias, and facilitate the investigation of micro-processes affecting behavior in real-world settings. Studies using EMA evaluate specific phenomena or experiences in the research participants’ lives or trace the participants at regular points of time, typically through random time sampling, *via* technologies including telephones and written diaries to physiological sensors and electronic diaries (Shiffman et al., 2008). The potential of EMA has recently drawn the attention of researchers in the SLA field, and specifically in the area of L2 emotional research (see Elahi Shirvan et al., 2020; Derakhshan, 2022). Thus, a review of these potentials can contribute to deeper insights into how this method can be applied in future research in L2 emotional domain. The distinctive features of EMA and its benefits for researchers will be reviewed here, and then more specifically for the second language acquisition (SLA) domain. Afterwards, we can see how EMA studies can be fit for exploring the L2 emotional variables involved in the classroom language learning process. An exemplary work of research will be presented along with a summary of its findings and the main contributions similar studies could make to the SLA theory and practice.

## Distinctive features of EMA

The data collected through EMA can be used to answer research questions about specific events or contexts, individual differences, and the temporal variation of processes through time, and the interactions between the underlying constructs (Moskowitz and Young, 2006). Thus, they can represent both the complexity and the richness of the data obtained from EMA. The different forms of EMA methods share several distinctive features, which are summarized by Stone and Shiffman (1994) and Stone et al. (2007a) and presented below. EMA deals with data collected from real-world contexts, as the research participants live their natural lives. The ecological dimension of EMA exactly implies this because EMA helps to make a generalization about the participants’ real life, and accounts for the ecological validity (Shiffman, 2007). Another distinctive feature is that the measurements are made on the participants’ present condition (Shiffman et al., 2008). For instance, the self-reports in EMA enquire about the feelings an individual has at the current time, instead of looking for recalls or an abstraction of what already happened or how the individual felt at long intervals. The momentary dimension of EMA exactly means that it hopes to compensate for the bias and error that can occur in the retrospection method. In EMA, the units of assessment are selected strategically to be moments, whether according to random sampling (to represent the participants’ experiences through representative sampling) or the specific variables of interest (e.g., occasions when the individuals get distracted, get bored or enjoy themselves), or by any other type of sampling. The participants are supposed to take part in several measurements at several points of time, illustrating how their behavior and experiences change through time and in different circumstances.

As previously mentioned, EMA can include different methods (e.g., time-based design, event-based monitoring). What these all share is doing measurements of individuals’ recent or current conditions, repeatedly sampled through time, in their ecological and natural surroundings (Delespaul, 1995).

## Fundamental literature on EMA

The beginning of EMA is traced back to 1994 (Stone and Shiffman, 1994), and has continued to be an active research area for years. There has been a plentiful volume of research works relying on EMA methodologies. The popularity of EMA as a helpful method of research is evident by the existing reviews and books published on this topic, such as the books about EMA-related methods and the reports published by Stone et al. (2007b), Hektner et al. (2007), and Fahrenberg and Myrtek (2001) employment of EMA uses for psychology by DeVries (1992), and data analysis discussions by Walls and Schafer (2006). There were some review studies by Wheeler and Reis (1991) about the sampling designs of EMA techniques, Scollon et al. (2003) about the advantages and disadvantages of EMA methods, Bolger et al. (2003) about the different applications of the diary technique, and Piasecki et al. (2007) about the uses of EMA techniques for clinical psychology.

Various studies have used EMA approaches for topics related to clinical psychology, personality, and health care domain, and a number of review articles elaborated on their use in some other areas (Tennen et al., 2005). The use of EMA methods in clinical psychology was discussed by Thiele et al. (2002). The applications of EMA in psychopharmacology were discussed by Moskowitz and Young (2006). In industrial psychology, the applications were discussed by Beal and Weiss (2003) to industrial psychology. The popularity of EMA methods lies in the fact that it can explore a wide array of experiences, conditions and behaviors.

Thiele et al. (2002) reviewed the published studies on diary keeping, and reported many academic works of research on mood, pain, anxiety disorders, eating, sleep, alcohol consumption and physical problems. Yet, the list of EMA studies is longer than that, as it is extended to investigating social support, depression, work activity, relationship initiation, psychotherapy, satisfaction, adverse effects of medications, psychological stress, and self-esteem.

The psychological issues investigated *via* EMA include anxiety disorders, bipolar disorder, addictive disorders, schizophrenia, depression, and ADHD and actually many cases of psychopathology (Colombo et al., 2020). Besides the clinical symptoms and syndromes, EMA has been also extensively employed to investigate the fundamental adaptation mechanisms related to adjustment, including social support, self-esteem and coping along with behaviors at the core of behavioral medicine and health psychology. To cut it short, EMA approaches have been employed to explore many psychological variables outside the second language acquisition (SLA) domain. Thus, they are worth being used in exploring the psychological traits involved in language learning too, as justified below.

## EMA major categories

There are two major categories of EMA depending on the arrangement, scheduling, and time coverage of analysis. In global measurements including personality surveys, the researcher presumes that the analysis covers the individual participant’s whole experience in single-shot data collection designs, losing sight of the dynamic and the developmental nature of the variable of interest. In EMA, moments or periods of time are assessed, which raises the problem of how to make sure that the periods or moments that are analyzed adequately represent the participant’s experience (see Shiffman et al., 2008). Occasionally, the analyses can be viewed as representative of the participant’s behavior or experience (Kop et al., 2001). Therefore, conducting an EMA study can fundamentally lead to a sampling design to capture moments in a subject’s life. The major effect on the design should be the purpose of the research.

In assessment and sampling designs, EMA may be typically categorized into the time-based sampling and event-based sampling designs (Wheeler and Reis, 1991; Shiffman, 2007). Time-based sampling often seeks to describe an experience more inclusively and broadly, for instance by observing the temporal changes in an emotion through a course with no pre-determined emphasis on individual events. Event-based designs do not aim to describe an individual’s whole experience but instead to emphasize specific separate episodes or events in individuals’ life excitement, trauma, shock, and plan the collection of data in relation to these episodes. These will be discussed in more detail below.

In some problematic conditions, the interest of research lies in specific episodes or events such as the instances of mental distraction, aggression, and panic attacks. Such instances can appropriately be investigated through event-based monitoring, which entails the triggering of assessments by the incidence of a predetermined event of interest to the research. For instance, the participants can be requested to do some rating whenever they experience a panic attack (Taylor et al., 1990), get involved in a social interaction that lasts for more than 10 min (Reis and Wheeler, 1991), or feel a special extreme emotion. Usually, the participants themselves decide when the event has happened and begin the assessment (despite the fact that some events may be detected automatically by certain instruments; see Kop et al., 2001). These designs need unambiguous descriptions of the event.

Time-based designs are fit for certain clinical phenomena, including pain and mood whose variation is continuous and is not simply captured in an episodic model of conceptualization. Sometimes, the event or the variable of interest can be continuously traced. In some others, this may not be probable, and EMA designs prefer time-based sampling. There are different types of time-based sampling designs depending on the time-plan, frequency, and schedule (see Delespaul, 1995). The resolution of the study will have been determined by the frequency of time-based evaluations. The required resolution is a function of the purpose of study, the existing knowledge about the target behavior, and the theoretical model of the research. Different time-based assessment plans are included in EMA. Some, like time series analysis and simple autocorrelation analysis, involve assessments set at fixed intervals, which lets the time block act as the analytic unit and provides evidence for analyses which need evenly timed evaluations. A special case is the use of daily diaries as already mentioned. Some works of research have applied somehow irregular time spaces, usually characterized by social variables. Also, sometimes, combined designs are used to test different research hypotheses (Shiffman et al., 2008).

## Ecological and dynamic approach to L2 emotional factors

The present study contends that the progress of L2 students’ emotional factors need to be investigated from a dynamic and ecological point of view. The emotions involved in language learning develop out of a network of relations between the L2 learner with the teacher and peers, who are all involved in the immediate environment for learning marked by emergent values, inherent dynamicity, activity, multiplicity and variability (Van Lier, 2004).

In this dynamic and ecological view, the association between the student and all the linguistic and non-linguistic factors (e.g., cognitive, emotional) that reside in the ecology of classroom become important (Elahi Shirvan et al., 2021; Mercer, 2021; Liu et al., 2022). Therefore, the dynamic and ecological investigation of language learners’ emotional variables reflects the connections between L2 learners and whatever is present around them and can, thus, offer new insights into how the affordances or agents contribute to the development or intensification of a particular emotional factor (e.g., enjoyment, boredom, grit, etc.). It also has the advantage of considering the learners’ surrounding environment significantly influential in the growth of different emotional states (Drew and Heritage, 1992). As pinpointed by Larsen-Freeman (2016), it is not possible to effectively explain the teaching or learning without reference to the contexts with which they are affiliated (Larsen-Freeman, 2016; Larsen Freeman, 2019).

Within an L2 classroom, Larsen-Freeman (2016) maintains that the constituent elements are not just the agents (i.e., instructors and learners and all their thoughts, feelings, performance, and actions) but the physical and temporal qualities of the learning context environment are also important. The time of class, the physical properties and everything about the space and time can significantly influence teaching and learning. Therefore, to explore the development of the emotions emerging out of the language learning experience, we should examine it as embedded within all these realities of the learning environment. That is why an ecological approach is relevant here. It considers the major contextual elements either human or not human that can all somehow affect the development and change in language learners’ patterns of emotional variables (Russell and Gajos, 2020).

We recurrently mentioned the notion of the emergent nature of emotions or emotional variables. According to Van Lier (2004), the seemingly over-emphasis on emergence is because language acquisition takes place when simple components are integrated to comprise a larger system. As approached by Larsen-Freeman (2016), emergence means something new appears when not anticipated out of a whole interconnected relationships among the constituent elements (of a whole). Thus, investigating EFL learners’ emotional variables and emotions from a dynamic and ecological point of view better reveals how the different interactive elements at different situated levels can lead to the emergence of a certain emotion. As for variability and diversity involved in the ecology of language learning, the implication is that teachers need to treat students differently and acknowledge their differences (Bourdieu, 1991; McLaren, 1998).

In sum, as individual L2 learners’ evident variability needs to be considered in the learning experience (Rose et al., 2013), a dynamic and ecological approach is deemed essential to explore language learners’ emotional variables. It offers a deeper understanding of how the patterns of a specific emotional variable (e.g., boredom, enjoyment, anxiety, etc.) may emerge differently across different learners. Following the procedure of an ecological approach and the presumed dynamic quality of language learners’ emotional variables (MacIntyre and Gregersen, 2012), the justification for using an EMA together with the complex dynamic system theory (CDST) is the emphasis of the two on the nuanced ecological elements (Van Lier, 2004) and operational mechanisms, emphasis on context and also the constituent elements of the L2 learning systems (Hiver and Al-Hoorie, 2016).

## Bridging the gap of the application of EMA for exploring L2 emotional variables

EMA has helped users to indicate that the emergent development of positive and negative affects results from modifying the proximal stimuli (Zohar et al., 2003; Fisher and Noble, 2004), personality-related variables (Grandey et al., 2002), and perceived job characteristics (Fisher, 2002). It is evident that the experiential quality of EMA allows users to adequately trace and confirm the dynamic mechanisms of actual affective conditions from an ecological point of view, and it makes contributions to theoretical underpinnings (Scollon et al., 2003). There is a significant dearth of studies using EMA in the SLA domain to investigate the dynamics of L2 emotions in the literature. There is an exemplary published work of research by Elahi Shirvan et al. (2020), which will be reviewed here. We will go on to emphasize that a frequent measurement of language learners’ emotions through time can add to our conceptualizing of the subjective quality of the L2 learners’ momentary feelings during multiple ecological timescales.

Inspired by the new change from negative psychology to positive psychology in SLA research (MacIntyre and Gregersen, 2012; MacIntyre and Mercer, 2014; Dewaele and Li, 2020), Elahi Shirvan et al. (2020) employed EMA for assessing L2 learners’ emotional variables. These researchers investigated the emergent patterns of language learners’ foreign language enjoyment in the SLA domain. They employed an ecological momentary assessment to add to the existing knowledge of the dynamics of this ecosystem in the network of individual students and their learning context. Elahi Shirvan et al. (2020), used a time-based sampling scheme of ecological momentary assessment and investigated the dynamic aspects of enjoyment in different points of time including seconds, minutes, etc. in an intermediate EFL program. They used open-ended interviews and also recorded journals for weeks. The findings of this study were useful as they revealed variation in the sequence of time scales, from moment-to-moment variation to the monthly changes. The researchers discussed the emergent patterns of enjoyment from one timescale to another according to the features of the CDST.

This exemplary work of research managed to highlight how an ecological and dynamic perspective managed to increase knowledge of the dynamics of an L2 learning-related emotion and reveal the emergent dynamic patterns of that emotion in several ecological timescales. As Elahi Shirvan et al. (2020) suggested, the use of EMA to assess the dynamics of foreign language enjoyment can be further developed in future investigations through an event-based sampling design. Yet, it is noteworthy that scrutinizing the dynamics of the L2 emotion they explored (i.e., foreign language enjoyment) and its ecological time-dependent changes is still in its infancy. Exploring these dynamics using EMA can reveal the context-bound quality of the emotion system. Overall, building on the findings of this study and other similar works of research can lead to the development of a representative model of L2-related emotions and their dynamic nature.

As for pedagogical implications, the findings of EMA studies of L2 emotions can probably show that even similar L2 students can experience diverse degrees of emotions in diverse time-points of an L2 course. Teachers can be made aware that, in spite of the self-directed factors contributing to L2 learners’ emotions, they can have a major role in the students’ points of time of positive emotions and probably in moments they may feel some negative emotion. Thus, the significance of teacher’s role is highlighted more than ever before, as their role is more determining in the development of a supportive and positive class climate. Furthermore, teachers need to be made aware that the effectiveness of their role in the emotional experiential moments of the language learners is not always the same, and that they need to be particularly careful about and aware of the self-related factors that account for the private emotional zone that the students have (Elahi Shirvan et al., 2020).

There are still many positive and negative L2 emotions that await being assessed *via* EMA in SLA studies. Examples of the former (positive emotions) are playfulness, L2 grit, passion for learning, compassion, and examples of the latter are foreign language learning boredom, L2 anxiety, and stress. These can be explored in EMA studies with the aim of answering this sample research question: How do moments of [L2 emotion of interest] vary under the impact of ecological elements in various moments?

EMA studies of these emotional variables enjoy the benefits of a dynamic and ecological perspective. Researchers who used traditional measurement methods for these emotions faced the problem that language learners either significantly overestimated or underestimated the emotions, cognitions, and behaviors they experienced before in their recalls after a long time (e.g., Thomas and Diener, 1990; Robinson and Clore, 2002). Also, as observed, the traditional notes on recollections were typically distorted by different conditions of reporting during the assessment, consisting of a temporary mood, a prevailing and frequent experience, or the most new one (Brief et al., 1995). Gathering moment-by-moment data several times, close to the time when an emotion rises, makes EMA an appropriate approach to address the distortions and biases related to the retrospective reports of L2 emotions (Smyth and Stone, 2003).

## Conclusion

The existing gap in the SLA research of L2-related emotions using EMA points to the yet-to-mature perceived value of the ecological and dynamic approach to investigating different emotional variables in the SLA domain. Though in the current research on L2 emotional factors, there is the recurrent emphasis on the social embeddedness of student (or instructor) related emotional factors (Bronfenbrenner, 1979, 1993), the ecological and dynamic momentary assessments of L2 emotions are scarce. The findings of the previous studies on L2 emotional constructs point to the socio-culturally constructed nature of language learners’ development of different emotions such as anxiety and enjoyment inside the interactive network of external and internal personal and contextual variables. Mapping out the moment-by-moment emergent patterns of the dynamic quality of language learners’ emotional variables in terms of values, quality, diversity, variability, and activity inside the ecology of the class helps to reveal useful outcomes on a multi-systemic scale.

There is research evidence in the SLA domain that several factors are involved in the development of L2 emotions, such as the language learners’ motivation, beliefs and, linguistic and cognitive factors in affecting the development of the students’ emotional variables (see Dörnyei and Ryan, 2015). Besides, L2 emotions are affected by L2 learners’ prior learning experience and attendance in extracurricular tasks. Moreover, such factors as classroom setting, the existing curriculum, course evaluation are effective in the emergence of L2 emotions. The key role of social, cultural and educational factors should not be ignored either. Thus, it is expected that EMA studies be proceeded by more extensive and in-depth follow-up studies to identify the roots of the wider range of underlying factors. The body of research on learner or teacher-related emotional variables showed that L2 emotions emerge dynamically from an interactive network of cognitive, linguistic, and emotional constructs. It can be also concluded that the contextual factors of the language teaching or learning processes cannot be wholly predictive of the future events. Therefore, the generalizability of the findings to other cases in other contexts should be made with caution (Ricca, 2012).

### Pedagogical implications and suggestions for further research

EMA studies enjoy distinctive benefits over conventional research methods of L2 emotional variables. EMS studies employ both an ecological and a dynamic approach to the exploration of emotional variables, which have a developmental nature. Despite the advantages and promises and extensive use in other fields of study such as clinical psychology, it has been still used significantly less in SLA studies. Considering the illuminating findings of the ecological studies of the SLA domain, it is hoped that the line of research especially the EMA types will continue to shed light on more emotional variables that have not yet been explored ecologically in language studies. Examples of these less explored L2 emotional variables are foreign language learners’ boredom, passion for learning, perceived loneliness and compassion.

Using time-based scales of the EMA framework to explore the dynamics of an L2 emotional variable can be followed up by future researchers in the form of event-based sampling scales. Yet, admittedly, understanding the dynamics of L2 emotions and their ecological temporal momentary changes is still rare. Investigating these dynamics using EMA can unravel the situation-specific nature of the L2 emotion system. Generally speaking, the findings obtained from the EMA studies can help to gradually build up an adequately representative model of L2 emotions and their dynamic developmental process. The findings of the EMA line of inquiry into L2 emotions can potentially prove the fact that even a single L2 learner may feel different intensities of a certain emotion in several distinctive scales of time during a language learning course. These can range from classroom interaction at the micro scale to the whole course at the macro scale.

The findings of the limited time-scaled studies revealed interesting details about the nuances of variation in the trajectory of the target variable (e.g., momentarily or monthly). More similar inquiries are needed to be capable of tracing the changes in the variables of interest more closely and realistically. Despite the growing interest in positive psychology, because there is a dearth of ecological research in SLA, it is suggested that both positive and negative emotional variables (involved in language learning) be explored using the EMA of ecological systems.

Regarding the limitation of EMA for the exploration of the dynamics of both positive and negative emotions in the field of SLA, it is worth noting that the procedures of data collection might seem time consuming and involve personal interventions. However, several techniques for the establishment of the trustworthiness of future studies using EMA in terms of credibility, transferability, dependability, and confirmability should be taken into account. Some of these techniques are prolonged engagement, persistent observation, thick description, and member-checking (see Lincoln and Guba, 1985).

## Author contributions

The author confirms being the sole contributor of this work and has approved it for publication.

## Funding

This study is sponsored by “2017 Project of Young Excellent & Talent Scholars” of University of Science and Technology Beijing. It is part of the outcomes of the project “Research on flipped classroom of college English education based on pan learning strategy” (Project No. 06200041), which is sponsored by Central higher education fundamental research funds. It is also part of the outcomes of USTB “Massive Online Open Course” Project (Project No. KC2021ZXKF21). The study is also funded by The 14th Five-Year Planned Teaching Material of USTB (Project No. JC2022YB037).

## Conflict of interest

The author declares that the research was conducted in the absence of any commercial or financial relationships that could be construed as a potential conflict of interest.

## Publisher’s note

All claims expressed in this article are solely those of the authors and do not necessarily represent those of their affiliated organizations, or those of the publisher, the editors and the reviewers. Any product that may be evaluated in this article, or claim that may be made by its manufacturer, is not guaranteed or endorsed by the publisher.
